# Risk factors for bloodstream infections due to carbapenem-resistant Enterobacterales: a nested case-control-control study

**DOI:** 10.1093/jac/dkae157

**Published:** 2024-07-11

**Authors:** Hongyu Zhou, Niccolò Buetti, Salvador Pérez-Galera, Jose Bravo-Ferrer, Belén Gutiérrez-Gutiérrez, María Paniagua-García, Jan Feifel, Julien Sauser, Tomi Kostyanev, Rafael Canton, Lionel K Tan, Dimitris Basoulis, Vicente Pintado, Emmanuel Roilides, Gorana Dragovac, Julian Torre-Cisneros, Deana Mediç, Murat Akova, Herman Goossens, Marc Bonten, Stephan Harbarth, Jesus Rodriguez-Baño, Marlieke E A De Kraker, COMBACTE- EURECA project team, Jesus Sojo-Dorado, Almudena de la Serna, Sophie Monteau, Virginia Palomo, Elena Soriano, David Gutiérrez, Elisa Moreno, Zaira Palacios, Isabel Morales, Natalia Maldonado, Jose Maria Reguera, Lucia Valiente de Santis, Antonio Plata Ciezar, Juan Diego Ruiz Mesa, Beatriz Sobrino Diaz, Ignacio Marquez Gomez, Ines Perez Camacho, Begoña Palop, Julian Torre-Cisneros, Angela Cano, Azahara Frutos-Adame, Julia Guzman-Puche, Irene Gracia-Ahufinger, Elena Perez-Nadales, Julian Torre-Gimenez, Athina Pyrpasopoulou, Elias Iosifidis, Elsa Chorafa, Biljana Carevic, Snezana Jovanovic, Ivana Radovanovic, Sladjana Petrovic, Slavica Cvetkovi, Lili Radulovic, Srdjan-Sanja Melentijevic, Natasa Miljkovic, Ana Perucica, Cenk Kirakli, Can Bicmen, Gunes Senol, Evelyn Shaw, Fe Tubau, Jordi Camara, Victor Daniel Gumucio, George L Daikos, John Deliolanis, Matthew E Falagas, Vassiliki Ch. Pitiriga, Nikolaos Triarides, Efstathia Argiti, Nikolaos J Legakis, Kyriakidou Margarita, Desirée Gijón-Cordero, Patricia Ruiz-Garbajosa, Amaya Suarez, Alessandro Bartoloni, Gian Maria Rossolini, Simin-Aysel Florescu, Maria Nica, Serban Benea, Daniela Talapan, Adriana Hristea, Sanja Prijić Maričić, Anita Jovetic, Marija Zivanovic Milenkovic, Angel Asensio, Mireia Cantero Caballero, Lina M Parra Ramírez, Belen Ruiz-Antoran, Rocio Layunta-Acero, Belen Ruiz-Antoran, Volkan Korten, Hüseyin Bilgin, Ufuk Hasdemir, George N Dalekos, Aggelos Stefos, Efthymia Petinaki, Nikolaos Spyridis, Athanasios Michos, Francesco Giuseppe De Rosa, Rossana Cavallo, Nicola Petrosillo, Antonio Dicaro, Pierluigi Viale, Maria Paola Landini, Marta Luisa Ciofi degli Atti, Mileva Masanovic, Dusan Matkovic, Dragan Satic, Milena Lopicic, Sotirios Tsiodras, Loukia Zerva, Francesco Blasi, Marta Di Pasquale, Milena Arghittu, Claudio Viscoli, Daniele Roberto Giacobbe, Anna Marchese, Andrei Vata, Olivia Dorneanu, Perlat Kapisyzi, Silva Tafaj, Adriana Vince, Arjana Tambic Andrasevic, Iva Butic, Evdoxia Tsigou, Alexandra Gavala, Theodora Biniari, Efstratios Maltezos, Apostolos Komnos, Spyros Karagiannis, Maria Tsalakou, Ioanna Voulgaridi, Charalampos Gogos, Iris Spiliopoulou, Fabio Franzetti, Sara Rimoldi, Massimo Antonelli, Gennaro De Pascale, Valentina Di Gravio, Teresa Spanu, Mihaela Lupse, Mirela Flonta, Dan Corneci, Mariana Buzea, Dana Tomescu, Alexandra Marcu, Camelia Ghita, Anca Georgescu, Leonard Azamfirei, Edit Székely, Goran Mitrović, Ljiljana Bukarica, Teodora Vitorovic, Nataša Lukić Krstić, Goran Mitrovic, Branislava Kocic, Marina Dinic, Lul Raka, Arsim Kurti, Beatriz Díaz-Pollán, Belen Loeches, Jose Ramón Arribas López, Julia Origüen Sabater, Fernando Chaves, Patricia Muñoz, Alpay Azap, Ceren Karahan, Banu Sancak, Arife Sahin, Halis Akalin, Cüneyt Ozakin

**Affiliations:** Infection Control Program, Geneva University Hospitals and Faculty of Medicine, Geneva, Switzerland; Department of Hospital Infection Control, The First Affiliated Hospital of Chongqing Medical University, Chongqing, China; Infection Control Program, Geneva University Hospitals and Faculty of Medicine, Geneva, Switzerland; Infection Antimicrobials Modeling Evolution (IAME) U 1137, INSERM, Université Paris-Cité, Paris, France; Unidad Clínica de Medicina Interna, Hospital Universitario Virgen Macarena, Seville, Spain; Instituto de Biomedicina de Sevilla (IBIS)/CSIC/Departamento de Medicina, Universidad de Sevilla, Seville, Spain; Instituto de Biomedicina de Sevilla (IBIS)/CSIC/Departamento de Medicina, Universidad de Sevilla, Seville, Spain; Unidad Clínica de Enfermedades Infecciosas y Microbiología, Hospital Universitario Virgen Macarena, Seville, Spain; Instituto de Biomedicina de Sevilla (IBIS)/CSIC/Departamento de Medicina, Universidad de Sevilla, Seville, Spain; Unidad Clínica de Enfermedades Infecciosas y Microbiología, Hospital Universitario Virgen Macarena, Seville, Spain; CIBER de Enfermedades Infecciosas (CIBERINFEC), Instituto de Salud Carlos III, Madrid, Spain; Instituto de Biomedicina de Sevilla (IBIS)/CSIC/Departamento de Medicina, Universidad de Sevilla, Seville, Spain; CIBER de Enfermedades Infecciosas (CIBERINFEC), Instituto de Salud Carlos III, Madrid, Spain; Department of Infectious Diseases, Microbiology and Parasitology, Virgen del Rocío University Hospital, Seville, Spain; Institute of Statistics, Ulm University, Ulm, Germany; Infection Control Program, Geneva University Hospitals and Faculty of Medicine, Geneva, Switzerland; Laboratory of Medical Microbiology, University of Antwerp, Antwerp, Belgium; National Food Institute, Technical University of Denmark, Lyngby, Denmark; CIBER de Enfermedades Infecciosas (CIBERINFEC), Instituto de Salud Carlos III, Madrid, Spain; Servicio de Microbiología, Hospital Universitario Ramón y Cajal and Instituto Ramón y Cajal de Investigación Sanitaria, Madrid, Spain; Research and Development, GlaxoSmithKline, London, UK; First Department of Internal Medicine, Laiko General Hospital, Athens, Greece; CIBER de Enfermedades Infecciosas (CIBERINFEC), Instituto de Salud Carlos III, Madrid, Spain; Servicio de Enfermedades Infecciosas, Hospital Universitario Ramón y Cajal and Instituto Ramón y Cajal de Investigación Sanitaria (IRYCIS), Madrid, Spain; Faculty of Health Sciences, Hippokration General Hospital of Thessaloniki, School of Medicine, Aristotle University of Thessaloniki, Thessaloniki, Greece; Faculty of Medicine and Institute of Public Health of Vojvodina, University of Novi Sad, Novi Sad, Serbia; CIBER de Enfermedades Infecciosas (CIBERINFEC), Instituto de Salud Carlos III, Madrid, Spain; Departamento de Ciencias Médicas y Quirúrgicas, Servicio de Enfermedades Infecciosas Hospital Universitario Reina Sofía/Instituto Maimónides de Investigación Biomédica de Córdoba (IMIBIC)/Universidad de Córdoba, Córdoba, Spain; Faculty of Medicine and Institute of Public Health of Vojvodina, University of Novi Sad, Novi Sad, Serbia; Department of Infectious Diseases and Clinical Microbiology, Hacettepe University Faculty of Medicine, Sihhiye, Ankara, Turkey; Laboratory of Medical Microbiology, University of Antwerp, Antwerp, Belgium; Julius Center for Health Sciences and Primary Care and University Medical Center Utrecht, Utrecht University, Utrecht, Netherlands; Infection Control Program, Geneva University Hospitals and Faculty of Medicine, Geneva, Switzerland; Instituto de Biomedicina de Sevilla (IBIS)/CSIC/Departamento de Medicina, Universidad de Sevilla, Seville, Spain; Unidad Clínica de Enfermedades Infecciosas y Microbiología, Hospital Universitario Virgen Macarena, Seville, Spain; CIBER de Enfermedades Infecciosas (CIBERINFEC), Instituto de Salud Carlos III, Madrid, Spain; Infection Control Program, Geneva University Hospitals and Faculty of Medicine, Geneva, Switzerland

## Abstract

**Background:**

Carbapenem-resistant Enterobacterales (CRE) bloodstream infections (BSIs) are a major threat to patients. To date, data on risk factors have been limited, with low internal and external validity. In this multicentre study, risk factors for CRE BSI were determined by comparison with two control groups: patients with carbapenem-susceptible Enterobacterales (CSE) BSI, and patients without Enterobacterales infection (uninfected patients).

**Methods:**

A multicentre, case-control-control study was nested in a European prospective cohort study on CRE (EURECA). CRE BSI:CSE BSI matching was 1:1, CRE BSI:Uninfected patients matching was 1:3, based on hospital, ward and length of stay. Conditional logistic regression was applied.

**Results:**

From March 2016 to November 2018, 73 CRE BSIs, 73 CSE BSIs and 219 uninfected patients were included from 18 European hospitals. For CRE versus CSE BSI, previous CRE colonization/infection [incidence rate ratio (IRR) 7.32; 95% CI 1.65–32.38) increased the risk. For CRE versus uninfected controls, independent risk factors included: older age (IRR 1.03; 95% CI 1.01–1.06), patient referral (long-term care facility: IRR 7.19; 95% CI 1.51–34.24; acute care hospital: IRR 5.26; 95% CI 1.61–17.11), previous colonization/infection with other MDR organisms (MDROs) (IRR 9.71; 95% CI 2.33–40.56), haemodialysis (IRR 8.59; 95% CI 1.82–40.53), invasive procedures (IRR 5.66; 95% CI 2.11–15.16), and β-lactam/β-lactamase inhibitor combinations (IRR 3.92; 95% CI 1.68–9.13) or third/fourth generation cephalosporin (IRR 2.75; 95% CI 1.06–7.11) exposure within 3 months before enrolment.

**Conclusions:**

Evidence of previous CRE colonization/infection was a major risk factor for carbapenem resistance among Enterobacterales BSI. Compared with uninfected patients, evidence of previous MDRO colonization/infection and healthcare exposure were important risk factors for CRE BSI. Targeted screening, infection prevention and antimicrobial stewardship should focus on these high-risk patients.

## Introduction

Bloodstream infections (BSIs) are among the most severe hospital-acquired infections, with Enterobacterales being the most frequently identified causative pathogens. Increases in resistance rates of Enterobacterales, especially to carbapenems, progressively complicate treatment strategies of infected patients.^1^ In Europe, in 2015, it was estimated that more than 2000 patients died because of carbapenem-resistant Enterobacterales (CRE) infections, mostly BSIs, and the number of attributable deaths increased more than six times from 2007 to 2015.^2^

Effective strategies to prevent or reduce the number of CRE BSIs are essential; however, to date, no harmonized strategy for CRE BSI prevention is available and data on modifiable risk factors are scarce.^3^ Most risk factor studies have been small, single-centre studies with a retrospective design.^4–7^ Moreover, these studies often selected patients with BSIs due to carbapenem-susceptible Enterobacterales (CSE) as controls. However, directly comparing CRE versus CSE can introduce selection bias, resulting in falsely identifying antibiotics as risk factor, or overestimating the OR of the resistance-defining antibiotic.^8–10^ The best way to overcome this bias is to include two control groups: patients with CSE, and patients without Enterobacterales infection (uninfected patients).

This study was part of the EUropean prospective cohort study on Enterobacteriaceae showing REsistance to CArbapenems (EURECA).^11^ Through a nested, matched case-control-control study, we determined risk factors for (i) carbapenem resistance among hospitalized patients with Enterobacterales BSI, and (ii) CRE BSI among uninfected, hospitalized patients in six European countries.

## Methods

### Ethics

The study was approved by the Ethics Committee of the Hospital Universitario Virgen Macarena (FIS-ATB-2015-01). The need to obtain written informed consent was waived due to the observational and epidemiological nature of the study. Approval was also gained at the participating centres according to local requirements. The study was conducted according to the principles of the Declaration of Helsinki and in accordance with the Medical Research Involving Human Subjects Act (WMO) and local guidelines in the participating countries.

### Study design

**Figure 1.**
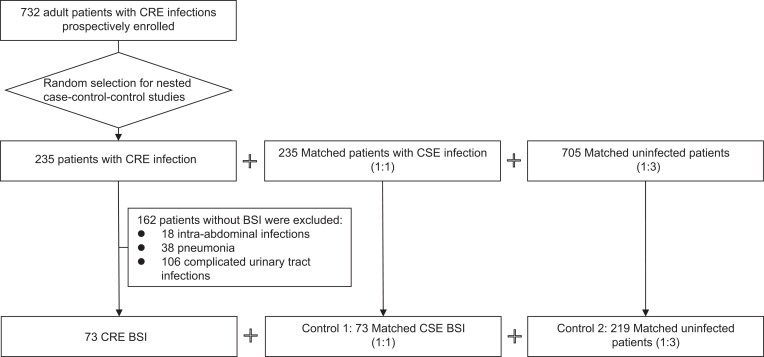
Flow chart for patient enrolment in EURECA between March 2016 and November 2018, including the final selection for the nested case-control-control study focusing on risk factors for carbapenem-resistant Enterobacterales (CRE) bloodstream infections (BSIs). CSE, carbapenem-susceptible Enterobacterales.

EURECA (trial registration number: NCT02709408) is a prospective, multinational, multicentre study that aims to characterize hospitalized patients with CRE infections in Europe.^11,12^ From March 2016 to November 2018, a cohort of 732 adult patients with CRE infections were enrolled. For a nested case-control-control study, additional CSE-infected patients and uninfected patients were recruited, for a randomly selected subset of 235 patients, matched 1:1 to CSE-infected, and 1:3 to uninfected patients, to be able to assess at least 20 risk factors (Figure 1). Our study focused on the subpopulation of 73 adult hospitalized patients with CRE BSI, and their matched controls (73 CSE BSI and 219 uninfected controls) (Figure 1), which provided a large enough sample size to simultaneously include around seven risk factors. Matching variables were hospital, type of hospital service and length of stay (LOS) before CRE BSI of the case (minus 0–3 days, or minus 0–7 days if LOS of CRE >14 days, or LOS of minimum 30 days if LOS of CRE >30 days). For matching of CRE:CSE, type of acquisition (community or nosocomial) and source of bacteraemia were considered as well. All patients were followed for a period of 30 days after inclusion.

### Setting

Fifty hospitals participated in EURECA from 10 countries across Europe. Sites were selected based on rates of infection due to CRE, clinical and laboratory capacity, and experience in clinical studies. Patients selected for the current study were enrolled from 18 hospitals, in Italy (*n* = 5), Spain (*n* = 4), Serbia (*n* = 4), Greece (*n* = 3), Romania (*n* = 1) and Turkey (*n* = 1).

### Inclusion and exclusion criteria

Patients were included if they met the following criteria: (i)  ≥ 18 years; (ii) signed informed consent form if requested by the local Institutional Review Board. Additionally, for patients with Enterobacterales BSI: (iii) patients had an Enterobacterales BSI, defined as a positive blood culture with isolation of CRE or CSE in patients fulfilling systemic inflammatory response syndrome criteria^13^ of sepsis, and for uninfected patients: (iv) patients without Enterobacterales infection during the selected hospitalization.

Exclusion criteria included: (i) patients with do-not-resuscitate orders or with a life expectancy of <30 days. Additional exclusion criteria for patients with Enterobacterales BSI: (ii) the infection was considered to be polymicrobial according to standard microbiological interpretations of culture results; (iii) participation in a trial that included active treatment for Enterobacterales BSI; and (iv) previously included in the EURECA CRE cohort.

### Data collection

Data were collected by dedicated onsite investigators in each of the sites, through a standardized, electronic case report form, with internal validity checks to improve data quality. Data consistency and completeness were checked regularly and issues were resolved through integrated patient-specific queries. Data included demographics, hospital admission characteristics, clinical characteristics, antimicrobial exposure, colonization status [MDR organisms (MDROs)] and microbiological characteristics. Possible CRE exposure risk was also recorded, and included travel, contact with animals, hospital contact, contact with CRE-colonized people, and evidence of previous infection/colonization by CRE. The latter is defined as confirmed CRE infection/colonization documented in the patient’s microbiological records (no time limit); if no previous CRE culture was recorded, it was considered as no evidence. For definitions see Table S1 (available as Supplementary data at *JAC* Online). Antimicrobial susceptibility was phenotypically and genotypically confirmed at a central laboratory in Antwerp, Belgium. STROBE recommendations for reporting results of observational studies were followed.

### Statistical analysis

Descriptive statistics are displayed separately for matched CRE BSI, CSE BSI and uninfected patients, summarized by median and IQR, or absolute numbers and proportions as appropriate. *P* values are based on conditional logistic regression to consider matching.

Analyses compared CRE BSI versus CSE BSI, and CRE BSI versus uninfected controls. All clinically relevant variables were analysed with univariable conditional logistic regression to determine their association with CRE BSI. For continuous variables, linearity was checked using likelihood ratio tests comparing models applying natural cubic splines versus a linear relationship; cut-offs were based on the lowest Akaike information criteria (AIC) values. All variables with *P* < 0.10 in the univariable analysis were considered for the conditional multivariable logistic regression. Collinearity was checked by the variance inflation factor; if needed, the clinically most relevant variable was selected. The final model was selected using the best subset method based on AIC values. Because missing data were sparse, complete case analysis was applied, the impact of which was assessed in sensitivity analyses. The coefficients from the conditional logistic regression are interpreted as incidence rate ratios (IRRs), due to the specific study design—a case-control study nested in an open cohort, where controls were matched on LOS before enrolment of the case.^14^

Sensitivity analyses were performed to check stability of results: (i) we examined the impact of complete case analysis, applying worst-case (all patients with missing data were positive) and best-case scenarios (all patients with missing data were negative) for two variables with missing data ‘evidence of previous colonization/infection with CRE’ and ‘evidence of previous colonization/infection with other MDROs’; (ii) we determined risk factors specifically for hospital-associated CRE BSI.

A two-sided *P* < 0.05 was considered statistically significant; 95% CIs are reported. The analyses were performed using R software, version 4.1.0.

## Results

Overall, 73 case patients with CRE BSIs could be selected from the EURECA dataset, matched to 73 control patients with CSE BSI (CSE group) and 219 uninfected patients (Figure 1). All matches fulfilled the preset matching criteria, with seven minor exceptions for LOS before enrolment. For 6/292 (2%) controls LOS before enrolment was 1–2 days compared with CRE BSI on admission, whereas 1/73 (1.4%) CSE BSI controls had 11 days of stay before infection compared with a CRE case with 21 days (max. 7 days difference). As such, this variable was considered for multivariable analysis.

### Patients infected by CRE BSI versus CSE BSI

#### Patient characteristics

Most CRE and CSE BSI patients came from Spain (56/146), Greece (32/146) and Serbia (31/146); had a similar median age, 69.0 (IQR 59.5–77.0) years and 70.0 (IQR 59.0–80.0), respectively; were male [CRE: 40/73 (54.8%) and CSE: 38/73 (52.1%)], were overweight or obese [CRE: 38/69 (55.1%) and CSE: 41/72 (57.0%)], and were admitted from home [CRE: 50/73 (68.5%) and CSE: 60/73 (82.2%)]. Evidence of previous colonization/infection with CRE was significantly different between CRE and CSE patients [26.5% (18/68) versus 4.2% (3/72), respectively, *P* = 0.004). Exposures to carbapenems (28.8% versus 11.0%, *P* = 0.011) and antimicrobials only active against Gram-positive pathogens (30.1% versus 16.4%, *P* = 0.004) were significantly higher for CRE compared with CSE BSI as well (Table 1). Infection sources and microbiological characteristics are described in Table 2.

**Table 1.** Baseline characteristics of adult hospitalized patients with carbapenem-resistant bloodstream infections and matched controls with carbapenem-susceptible bloodstream infection, or without Enterobacterales infection, admitted to 18 European hospitals between March 2016 and November 2018

Variables	Patients with CRE BSI (*N* = 73)	Matched patients with CSE BSI (*N* = 73)	Matched patients without Enterobacterales infection (*N* = 219)	Comparison between CRE and CSE BSI patients *P* value^a^	Comparison between CRE and uninfected patients *P* value^a^
Demographic information					
Age, y, median (IQR)	69.0 (59.5–77.0)	70.0 (59.0–80.0)	64.0 (51.0–75.0)	0.639	0.009
Male sex, *n* (%)	40 (54.8)	38 (52.1)	131 (59.8)	0.732	0.447
BMI, kg/m^2^, median (IQR)^b^	25.5 (23.7–29.2)	25.4 (21.8–27.9)	25.1 (22.9–28.7)	0.182	0.977
BMI <25 kg/m^2^, *n* (%)	31 (44.9)	31 (43.1)	104 (48.1)	0.931	0.806
BMI 25–29 kg/m^2^, *n* (%)	27 (39.1)	31 (43.1)	73 (33.8)		
BMI ≥ 30 kg/m^2^, *n* (%)	11 (15.9)	10 (13.9)	39 (18.1)		
Country of origin, *n* (%)					
Spain	27 (37.0)	29 (39.7)	88 (40.2)	0.503	0.155
Greece	16 (21.9)	16 (21.9)	44 (20.1)		
Serbia	16 (21.9)	15 (20.5)	44 (20.1)		
Italy	7 (9.6)	7 (9.6)	15 (6.8)		
Turkey	3 (4.1)	2 (2.7)	9 (4.1)		
Romania	2 (2.7)	1 (1.4)	5 (2.3)		
Other	2 (2.7)	3 (4.1)	14 (6.4)		
Hospital admission characteristics, *n* (%)					
Patient referral					
Home	50 (68.5)	60 (82.2)	191 (87.2)	0.071	<0.001
Long-term care facility	9 (12.3)	4 (5.5)	11 (5.0)		
Another acute care hospital	14 (19.2)	9 (12.3)	17 (7.8)		
Emergency admission	2 (2.7)	3 (4.1)	13 (5.9)	0.657	0.206
Hospital service on enrollment^c^					
Medical	46 (63.0)	46 (63.0)	138 (63.0)	1.000	1.000
Surgical	13 (17.8)	13 (17.8)	39 (17.8)		
ICU	14 (19.2)	14 (19.2)	42 (19.2)		
Length of hospital stay before enrolment ≥1 d	51 (69.9)	48 (65.8)	141 (64.4)	0.327	0.023
Clinical characteristics					
Comorbidities					
Charlson comorbidity index, median (IQR)	2.0 (1.0–3.0)	2.0 (1.0–4.0)	2.0 (1.0–3.0)	0.683	0.203
Myocardial infarction, *n* (%)	9 (12.3)	9 (12.3)	17 (7.8)	1.000	0.230
Congestive heart failure: NYHA grade ≥2, *n* (%)	10 (13.7)	8 (11.0)	31 (14.2)	0.618	0.909
Peripheral artery disease, *n* (%)	14 (19.2)	12 (16.4)	46 (21.0)	0.618	0.713
Cerebrovascular disease, *n* (%)	5 (6.8)	5 (6.8)	10 (4.8)	1.000	0.402
Dementia, *n* (%)	6 (8.2)	7 (9.6)	7 (3.2)	0.763	0.090
Chronic pulmonary disease, *n* (%)	6 (8.2)	11 (15.1)	30 (13.7)	0.206	0.206
Connective tissue disease, *n* (%)	4 (5.5)	2 (2.7)	10 (4.6)	0.423	0.752
Ulcerative disease, *n* (%)	2 (2.7)	3 (4.1)	9 (4.1)	0.571	0.580
Mild liver disease, *n* (%)	5 (6.8)	2 (2.7)	9 (4.1)	0.215	0.336
Severe liver disease, *n* (%)	1 (1.4)	1 (1.4)	8 (3.7)	1.000	0.318
Diabetes mellitus without organ damage, *n* (%)	17 (23.3)	18 (24.7)	40 (18.3)	0.827	0.334
Diabetes with target organ damage, *n* (%)	5 (6.8)	10 (13.7)	15 (6.8)	0.206	1.000
Hemiplegia, *n* (%)	4 (5.5)	3 (4.1)	4 (1.8)	0.706	0.120
Moderate or severe kidney disease, *n* (%)	15 (20.5)	8 (11.0)	31 (14.2)	0.083	0.108
Metastatic solid tumour, *n* (%)	3 (4.1)	5 (6.8)	8 (3.7)	0.484	0.847
Any tumour, not metastasic, *n* (%)	14 (19.2)	13 (17.8)	30 (13.7)	0.827	0.214
Leukaemia, *n* (%)	4 (5.5)	5 (6.8)	11 (5.0)	0.706	0.842
Lymphoma, *n* (%)	4 (5.5)	4 (5.5)	12 (5.5)	1.000	1.000
AIDS, *n* (%)	0 (0.0)	1 (1.4)	3 (1.4)	0.998	0.998
HIV infection with <200 CD4/mm^3^, *n* (%)	0/72 (0.0)	1/71 (1.4)	4/218 (1.8)	0.998	0.998
Immunosuppression^d^, *n* (%)	20 (27.4)	23 (31.5)	54 (24.7)	0.514	0.549
Invasive procedures within 3 mo before enrolment, *n* (%)	51 (69.9)	46 (63.0)	96 (43.8)	0.321	<0.001
Surgery during the previous month, *n* (%)	22 (30.1)	23 (31.5)	53 (24.2)	0.796	0.176
Endoscopic procedure in the week before enrolment, *n* (%)	5 (6.8)	8 (11.0)	12 (5.5)	0.372	0.623
CRE exposure risk, *n* (%)					
CRE exposure risk in community in the last 6 mo					
Travel abroad	2/71 (2.8)	1/73 (1.4)	17/218 (7.8)	0.571	0.175
Contact with pets at home	9/71 (12.7)	9/70 (12.9)	34/209 (16.3)	1.000	0.315
Frequent contact with livestock	0/71 (0.0)	0/72 (0.0)	5/212 (2.4)	—	0.997
Ambulatory contact with persons known to be colonized by CRE	6/57 (10.5)	2/56 (3.6)	4/177 (2.3)	0.998	0.998
CRE exposure risk in healthcare facility					
Patient worked as healthcare worker or caregiver during last year	1 (1.4)	0 (0.0)	2/215 (0.9)	0.998	0.741
Another patient/s with CRE in the same ward during present admission	29/70 (41.4)	27/69 (39.1)	86/217 (39.6)	0.514	0.579
Previous hospitalization during the last 6 mo	40 (54.8)	32 (43.8)	72 (32.9)	0.174	0.001
Nursing home or other long term-care facility residency during the last 6 mo	10 (13.8)	9 (12.3)	9 (4.1)	0.796	0.005
Chronic dialysis	8 (11.0)	5 (6.8)	14 (6.4)	0.372	0.140
Haemodialysis	7 (9.6)	5 (6.8)	10 (4.6)	0.530	0.061
Peritoneal dialysis	1 (1.4)	0 (0.0)	4 (1.8)	0.998	0.782
Evidence of previous colonization/infection with CRE	18/68 (26.5)	3/72 (4.2)	1/217 (0.5)	0.004	<0.001
Evidence of previous colonization/infection with other MDROs (MRSA, VRE, ESBL-producer)	12/71 (16.9)	7/71 (9.9)	14/217 (6.5)	0.147	0.005
Antimicrobial exposure within 3 mo before enrolment	53 (72.6)	48 (65.8)	123 (56.2)	0.339	0.008
Colistin	7 (9.6)	7 (9.6)	11 (5.0)	1.000	0.061
Aminoglycosides	8 (11.0)	6 (8.2)	13 (5.9)	0.566	0.131
Quinolones	21 (28.8)	15 (20.5)	50 (22.8)	0.277	0.298
Macrolides	2 (2.7)	3 (4.1)	4 (1.8)	0.657	0.640
Cephalosporins, first/second generation	9 (12.3)	13 (17.8)	20 (9.1)	0.292	0.339
Cephalosporins, third/fourth generation	20 (27.4)	15 (20.5)	38 (17.4)	0.321	0.043
Carbapenems	21 (28.8)	8 (11.0)	25 (11.4)	0.011	<0.001
β-lactam + β-lactamase inhibitor	28 (38.4)	21 (28.8)	44 (20.1)	0.183	0.001
Other β-lactam antibiotics	7 (9.6)	10 (13.7)	4 (1.8)	0.372	0.007
Active against Gram-positive only	22 (30.1)	12 (16.4)	28 (12.8)	0.004	0.001
Other	14 (19.2)	17 (23.3)	35 (16.0)	0.549	0.454

BSI, bloodstream infection; CRE, carbapenem-resistant Enterobacterales; CSE, carbapenem-susceptible Enterobacterales; MDROs, multidrug-resistant organisms; NYHA, New York Heart Association.

^a^Based on univariable conditional logistic regression, in case of categorical variables it refers to the likelihood ratio test.

^b^The denominators for patients with CRE BSI, CSE BSI and without Enterobacterales infection are 69, 72 and 216, respectively.

^c^Matching variable for patients with CRE BSI versus patients with CSE BSI and patients without Enterobacterales infection, respectively.

^d^Immunosuppression was defined as the receipt of solid organ transplantation, bone marrow/stem cell transplantation or immunosupressive drugs (including cancer chemotherapy, classic immunosuppressants, biologicals or steroids) within 3 mo before enrolment, or with neutropenia (<500 cells/mm^3^) on enrolment.

**Table 2.** Clinical and microbiological characteristics associated with bloodstream infection among adult hospitalized patients with carbapenem-resistant bloodstream infections (CRE-BSIs) and matched controls with carbapenem-susceptible bloodstream infections (CSE-BSIs) admitted to 18 European hospitals between March 2016 and November 2018

Variables	Patients with CRE BSI (*N* = 73)	Matched patients with CSE BSI (*N* = 73)	*P* value^a^
	*n* (%)	*n* (%)	
Type of BSI acquisition^b^			
Nosocomial	47 (64.4)	47 (64.4)	1.000
Community-onset healthcare-associated	20 (27.4)	20 (27.4)	
Strict community-acquired	6 (8.2)	6 (8.2)	
BSI sources^b^			
Urinary tract	27 (37.0)	27 (37.0)	1.000
Pneumonia	6 (8.2)	6 (8.2)	
Intra-abdominal	11 (15.1)	11 (15.1)	
Intravascular catheter	10 (13.7)	10 (13.7)	
Other	3 (4.1)	3 (4.1)	
Unknown source	16 (21.9)	16 (21.9)	
Pathogen			
*Escherichia coli*	5 (6.8)	30 (41.1)	<0.001
*Klebsiella pneumoniae*	60 (82.2)	25 (34.2)	
*Enterobacter cloacae*	3 (4.1)	7 (9.6)	
Other Enterobacterales^c^	5 (6.8)	11 (15.1)	
Carbapenemase producer among CRE	70 (95.9)		
Type of carbapenemase			
OXA-48	35 (50.0)		
KPC (2/3)	22 (31.4)		
NDM-1	6 (8.6)		
VIM (1/4)	3 (4.3)		
Two types identified^d^	4 (5.7)		

BSI, bloodstream infection; CRE, carbapenem-resistant Enterobacterales; CSE, carbapenem-susceptible Enterobacterales.

^a^Based on univariable conditional logistic regression.

^b^Matching variable.

^c^For patients with CRE BSI, other Enterobacterales included *Klebsiella* species not *K. pneumoniae* (*n* = 1), *Enterobacter* species not *E. cloacae* (*n* = 1), *Serratia* species (*n* = 1), *Proteus mirabilis* (*n* = 1) and *Citrobacter freundii* (*n* = 1). For matched patients with CSE BSI, other Enterobacteriaceae included *Klebsiella oxytoca* (*n* = 1), *Enterobacter aerogenes* (*n* = 1), *Serratia marcescens* (*n* = 3), *P. mirabilis* (*n* = 3), *Citrobacter* species (*n* = 1), *Morganella morganii* (*n* = 1) and *Providencia rettgeri* (*n* = 1).

^d^OXA-48 and NDM-1 (*n* = 2), KPC-2 and VIM-1 (*n* = 1), and KPC-3 and NDM-1 (*n* = 1).

#### Risk factor analysis

A total of 140/146 (95.9%) patients could be included in risk factor analysis. Patient referral, moderate or severe kidney disease, evidence of previous colonization/infection with CRE, and exposure to carbapenems and antimicrobials active against Gram-positive pathogens within 3 months before enrolment were selected in univariable analysis (Table S2). The final multivariable model included evidence of previous colonization/infection with CRE (IRR 7.32; 95% CI 1.65–32.38), and carbapenem exposure within 3 months before enrolment (IRR 2.76; 95% CI 0.95–7.99) as independent, significant risk factors for carbapenem resistance among Enterobacterales BSI.

### Patients with CRE BSI versus uninfected patients

#### Patient characteristics

In CRE BSI and uninfected controls, over half of the patients were male [40/73 (54.8%) and 131/219 (59.8%), respectively], and overweight or obese [38/69 (55.1%) and 112/216 (51.9%), respectively]. The median age of CRE BSI patients, 69.0 (IQR 59.5–77.0) years, was significantly higher than that of uninfected patients (64.0, IQR 51.0–75.0). CRE BSI patients compared with uninfected patients, were more often transferred from a long-term care facility [LTCF; 9/73 (12.3%) versus 11/219 (5.0%), *P* = 0.003] or another acute care hospital [ACH, 14/73 (19.2%) versus 17/219 (7.8), *P* = 0.001]. Contact with healthcare was more frequent for CRE BSI patients as well, including previous invasive procedures, hospitalization and LTCF residency, evidence of colonization/infection with other MDROs, and antimicrobial use. Overall, 18/68 (26.5%) of CRE BSI cases had evidence of previous colonization/infection with CRE versus only one uninfected patient [1/217 (0.5%), *P* < 0.001] (Table 1).

#### Risk factor analysis

A total of 288/292 (98.6%) patients could be included in risk factor analysis. In total, 14 variables were selected in the univariable analysis including age, patient referral, LOS before enrolment, dementia, invasive procedures, previous hospitalization, chronic haemodialysis, evidence of previous colonization/infection with other MDROs, and exposure to specific antimicrobials (Table S2). For multivariable analysis, LTCF residency, chronic dialysis and exposure to any antimicrobial were not included because of collinearity with patient referral, chronic haemodialysis and exposure to specific antimicrobials, respectively. Evidence of previous colonization/infection with CRE was not included in the multivariable model to prevent sparse-data bias (1/217 uninfected patients was positive).

In the final, multivariable model the independent risk factors for acquiring CRE BSI with the highest values included evidence of previous colonization/infection with other MDROs (IRR 9.71; 95% CI 2.33–40.56), chronic haemodialysis (IRR 8.59; 95% CI 1.82–40.53), patient referral (LTCF: IRR 7.19; 95% CI 1.51–34.24, another ACH: IRR 5.26; 95% CI 1.61–17.11), and invasive procedures within 3 months before enrolment (IRR 5.66; 95% CI 2.11–15.16) (Table 3).

**Table 3.** Multivariable, conditional logistic regression analysis (AIC = 141.77) for risk factors of carbapenem-resistant Enterobacterales bloodstream infection among adult hospitalized patients, by comparing cases with carbapenem-resistant bloodstream infections with matched controls without Enterobacterales infection admitted to 18 European hospitals between March 2016 and November 2018 (*n* = 288)

Variables	Incidence rate ratio (95% CI)	*P* value
Age, y	1.03 (1.01–1.06)	0.019
Patient referral		
Admission from home	Reference	
Transfer from long-term care facility	7.19 (1.51–34.24)	0.013
Transfer from another acute care hospital	5.26 (1.61–17.11)	0.006
Invasive procedures^a^	5.66 (2.11–15.16)	0.001
Chronic haemodialysis	8.59 (1.82–40.53)	0.007
Evidence of previous colonization/infection with other MDROs (MRSA, VRE, ESBL-producer)	9.71 (2.33–40.56)	0.002
β-Lactam + β-lactamase inhibitor exposure	3.92 (1.68–9.13)	0.002
Third/fourth-generation cephalosporin exposure	2.75 (1.06–7.11)	0.037

BSI, bloodstream infection; CRE, carbapenem-resistant Enterobacterales; MDROs, multidrug-resistant organisms.

^a^Central venous catheter, urinary catheter or mechanical ventilation within 3 mo before enrolment.

### Comparison of risk factors between the two multivariable models

Evidence of previous colonization/infection with CRE was a significant risk factor in both comparisons, strengthening the assumption that this is an important risk factor for CRE BSI. Carbapenem use, the resistance-defining antibiotic, was only identified as a risk factor (*P* = 0.062) in the comparison CRE versus CSE BSI, which means it should be considered as a spurious finding due to control group selection. In the comparison CRE BSI versus uninfected, many additional risk factors were identified, underlining the strong association between healthcare exposure and risk of Enterobacterales BSI in general, including chronic haemodialysis, transfer from LTCFs and antimicrobial use.

### Sensitivity analyses

In the risk factor model for CRE BSI versus CSE BSI, imputing missing data for ‘evidence of previous colonization/infection with CRE’, applying worst- or best-case scenario, carbapenem exposure within 3 months before enrolment changed from borderline to fully significant in both scenarios (Table S3). In the risk factor model for CRE BSI versus uninfected patients, imputing missing data for ‘evidence of previous colonization/infection with other MDROs’, applying worst- or best-case scenario, the significant variables remained the same (Table S4).

Focusing on hospital-associated CRE BSIs, chronic pulmonary disease was detected as an additional factor associated with decreased risk for carbapenem resistance among patients with Enterobacterales BSI (Table S5). For the comparison CRE BSI versus uninfected, evidence of previous colonization/infection with other MDROs was no longer included as a risk factor, whereas third/fourth-generation cephalosporin and other β-lactam antibiotic exposures were now borderline significant (Table S6).

## Discussion

### Main findings

In this prospective, European, multicentre, nested case-control-control study, a comprehensive set of patient characteristics could be compared between patients with CRE BSI, CSE BSI and without Enterobacterales infection. Our study validated prior hypotheses, while distinguishing risk factors associated with carbapenem resistance specifically and Enterobacterales infections in general. We identified evidence of previous colonization/infection with CRE as the most important risk factor for carbapenem resistance among Enterobacterales BSI in adult hospitalized patients. In addition, evidence of previous colonization/infection with other MDROs (MRSA, VRE, ESBL-producer), healthcare exposure risk, including chronic haemodialysis, invasive procedures within 3 months before enrolment, transfer from LTCF/ACH and exposure to antibiotics were important risk factors for Enterobacterales infections in general.

### Previous colonization/infection with CRE

Our study highlights the strong association between evidence of previous colonization/infection with CRE and subsequent CRE BSI, which was found in the comparison of CRE BSI cases with CSE BSI cases, as well as with uninfected controls. Analysis of risk factors for CRE infections in general (EURECA), also confirmed the importance of colonization.^12^ Other previous studies support the association between CRE colonization and CRE BSI among critically ill patients, mainly through comparison of CRE BSI versus uninfected patients.^6,15,16^ In patients with liver transplantation^15^ or haematological malignancies,^6^ effect estimates as high as 16.6 (HR) and 11.1 (OR) were identified. This highlights the significance of regular screening of high-risk patients for presence of CRE, especially considering that in this study only 27% of patients with CRE BSI were known CRE carriers, which included information from colonization/infection during previous hospitalizations. Other studies have found percentages as high as 54% colonization among patients developing CRE infection. In this study, 16/18 hospitals had active screening strategies for CRE, mostly for ICU patients (*n* = 12) and haematology, transplant or other high-risk patients (*n* = 7); some also screened transferred patients (*n* = 2), surgical patients (*n* = 2) or all patients (*n* = 1). The results of this study suggest that screening strategies might need to be extended to a larger patient population, whereby transferred patients especially would be an important target group. In addition to the patient population, the local epidemiological level of CRE plays a crucial role for active screening strategies. Only one assessment at admission could not be enough in hospitals with endemic CRE. Indeed, CRE carriage acquisition during hospital stay can occur and it is strongly associated with CRE BSI development in the following days.^17,18^

### Carbapenem exposure: a spurious risk factor

In previous studies with a case-control design, carbapenem exposure was a common risk factor for carbapenem resistance among adult hospitalized patients with Enterobacterales BSI.^4,5,19–22^ In the current study, using the case-control-control design, qualitative comparison of the two models indicated that the association between carbapenem use and carbapenem exposure was a spurious finding associated with control group selection bias.^10,23^ If patients with CSE BSI are selected as controls, it is unlikely that these patients would have had exposure to carbapenems, as this would have eliminated colonization by any CSE, and thus would have greatly reduced the likelihood of development of CSE BSI. Carbapenem exposure was not identified as a risk factor in the comparison between CRE BSI and uninfected patients, where this selection bias does not play a role, underlining the importance of control group selection in these types of studies.^8,9^ On the other hand, identification of risk factors may also depend on the mechanisms of carbapenem resistance considered. A multicentre case-control study in Singapore indicated that carbapenem exposure was an independent risk factor for non-carbapenemase-producing CRE versus carbapenemase-producing Enterobacteriaceae.^24^ This further supports the conclusion that carbapenem exposure was not a risk factor in this study, where 96% of CRE were associated with carbapenemase production.

### Study limitations

Our study has a few limitations. Firstly, despite recruiting from multiple healthcare facilities, only 73 CRE BSI cases could be enrolled, limiting the number of variables that could be considered in the multivariable model. Secondly, matching criteria for LOS before enrolment were not met for five controls, possibly confounding results. To mitigate this, LOS before enrolment was considered for risk factor analysis. Thirdly, for evidence of previous CRE colonization/infection, we had to depend on local screening and culturing practices. Nevertheless, this reflects the true daily practice. Finally, evidence of previous colonization/infection with CRE could not be included in the multivariable model for the comparison between CRE BSI and uninfected patients due to sparse-data bias;^25^ only one positive, uninfected patient was detected.

### Conclusions

In conclusion, based on a multicentre, matched, case-control-control study, which considered a variety of possible risk factors, evidence of previous colonization/infection with CRE was identified as a major risk factor for CRE BSI. For Enterobacterales BSI in general, other important high-risk patient characteristics include higher age, evidence of previous colonization/infection with other MDROs, or previous healthcare exposure, including chronic haemodialysis, invasive procedures during the past 3 months, transfer from LTCF/ACH, and specific antibiotic exposure. These risk factors should be considered to inform targeted screening and infection control measures to reduce CRE BSI risk. Novel approaches to successfully decolonize CRE carriers would be vital to further improve patient safety.

## Supplementary Material

dkae157_Supplementary_Data

## Transparency declarations

E.R. has served as consultant to Amplyx, Astellas, Gilead, MSD, Pfizer, Scynexis, GSK and Shionogi. J.T.-C. reports supports from MSD, Pfizer, Menarini and Shionogi, has served as consultant to MSD, participated on Advisory Boards from Shionogy and MSD, and reports travel supports from Gilead, Shionogy and Pfizer. L.K.T. is an employee of, and holds stocks and shares in, GlaxoSmithKline. M.A. reports research support from Pfizer and speaker fees for educational activities paid to the University accounts by Pfizer, Gilead and Sanofi. V.P. has participated in accredited educational activities sponsored by MSD, Pfizer and Shionogi and has been a consultant for Pfizer, Shionogi and Correvio. M.B. reports grants paid to his institution from Janssen Vaccines, Novartis, CureVac and Merck; participation in Advisory Boards with payment to his institution from Spherecydes, Pfizer, Merck and Astra-Zeneca, and participation in Data Safety Monitoring Boards with payment to his institution from Sanofi. All other authors have no conflicts to declare.

## Supplementary data

Tables S1 to S6 are available as Supplementary data at *JAC* Online.
