# Supplementary material for: Risk factors for bloodstream infections due to carbapenem-resistant Enterobacterales: a nested case-control-control study
Source: J Antimicrob Chemother. 2024 Jul 11;79(9):2132–41. doi: 10.1093/jac/dkae157 (PMC11368427; doi:10.1093/jac/dkae157)
Supplement: dkae157_Supplementary_Data [file dkae157_supplementary_data.docx]

**Risk factors for bloodstream infections due to carbapenem-resistant *Enterobacterales*: a nested case-control-control study**

Hongyu Zhou, Niccolò Buetti*, Salvador Pérez-Galera, Jose Bravo-Ferrer, Belén Gutiérrez-Gutiérrez, María Paniagua-García, Jan Feifel, Julien Sauser, Tomi Kostyanev, Rafael Canton, Lionel K. Tan, Dimitris Basoulis, Vicente Pintado, Emmanuel Roilides, Gorana Dragovac, Julian Torre-Cisneros, Deana Medić, Murat Akova, Herman Goossens, Marc Bonten, Stephan Harbarth, Jesus Rodriguez-Baño^#^, Marlieke E.A. de Kraker^#^ on behalf of the entire COMBACTE- EURECA project team.

^#^ Equal contribution

* Corresponding author:

Niccolò Buetti, MD, MSc, PhD

Infection Control Program, Geneva University Hospitals and Faculty of Medicine

Gabrielle-Perret-Gentil 4, 1205 Geneva, Switzerland

Phone: +41-795530072, Email: niccolo.buetti@hcuge.ch

**Contents**1. Table S1. Collected variables and study-specific definitions for carbapenem-resistant *Enterobacterales* bloodstream infection risk factor analysis……...……………………………….2

2. Table S2. Univariable analyses of risk factors for carbapenem-resistant *Enterobacterales* bloodstream infection among adult, hospitalized patients compared to matched controls with carbapenem-susceptible bloodstream infection, or without *Enterobacterales* infection……….4

3. Table S3. Sensitivity analysis for missing data for “evidence available for previous colonization/infection with carbapenem-resistant *Enterobacterales*” comparing risk factors for carbapenem-resistant versus carbapenem-susceptible *Enterobacterales* bloodstream infections……………..………………………………………………………………………………....7

4. Table S4. Sensitivity analysis for missing data for “evidence of previous colonization/infection with other multidrug-resistant organisms (methicillin-resistant *Staphylococcus aureus*, vancomycin-resistant *enterococci*, extended-spectrum beta-lactamase-producer)” comparing risk factors for carbapenem-resistant *Enterobacterales* bloodstream infection versus uninfected patients……………………………………................…8

5. Table S5. Sensitivity analysis for nosocomial or community-onset healthcare-associated CRE BSI comparing risk factors for carbapenem-resistant versus carbapenem-susceptible *Enterobacterales* bloodstream infections .................................................................................9

6. Table S6. Sensitivity analysis for nosocomial or community-onset healthcare-associated CRE BSI comparing risk factors for carbapenem-resistant *Enterobacterales* bloodstream infection versus uninfected patients.………………………………………..………………………10

7. References………………………………………………………………………………………….11

**Table S1. Collected variables and study-specific definitions for carbapenem-resistant *Enterobacterales* bloodstream infection risk factor analysis**

| Available variables | |  | Study-specific definitions for selected variables | |
| --- | --- | --- | --- | --- |
| Category | Variables |  | Selected variable | Definition |
| Case definition | CRE, CSE, BSI, uninfected patients, enrolment day |  | CRE | Any isolate identified as an *Enterobacterales* showing a MIC for meropenem or imipenem of ≥1 mg/L if using any dilution method, and/or ≤22 mm if using a disk-diffusion method (10 μg disks) |
|  |  |  | CSE | Any isolate identified as an *Enterobacterales* showing susceptibility to carbapenems according to the aforementioned criteria  Meropenem-susceptible and imipenem-susceptible isolates showing resistance to ertapenem were excluded. |
|  |  |  | BSI | Positive blood culture with isolation of CRE or CSE in patients fulfilling systemic inflammatory response syndrome (SIRS) criteria^1^ of sepsis |
|  |  |  | Uninfected patients | Patients without *Enterobacterales* infection during the selected hospitalization |
|  |  |  | Enrolment day | For patients with CRE and CSE BSIs, enrolment day was the collection date of the first blood sample positive for CRE or CSE, respectively. For uninfected patients, enrolment day was the date of inclusion in the study, whereby length of hospitalization before inclusion had to be matched to the correspondent CRE case. |
| Demographics | age, gender, body mass index, country of origin |  |  |  |
| Hospital admission characteristics | patient referral, emergency admission, hospital service on enrolment, length of stay before enrolment |  |  |  |
| Clinical characteristics | comorbidities based on the Charlson comorbidity index,^2^ HIV infection with <200 CD4 cells/mm^3^, immunosuppression, invasive procedures (central venous catheter, urinary catheter or mechanical ventilation) within 3 months before enrolment, surgery during the previous month, endoscopic procedure in the week before enrolment |  | Immuno-suppression | The receipt of solid organ transplantation, bone marrow/stem cell transplantation, or immunosupressive drugs (including cancer chemotherapy, classic immunosuppressants, biologicals, steroids) in the 3 months before enrolment, or with neutropenia (<500 cells/mm^3^) on enrolment |
| Possible CRE exposure risk factors | chronic dialysis, recent travel abroad, contact with pets or livestock, profession, contact with persons colonized by CRE, previous hospitalizations or long-term care facility residency, evidence of previous infection/ colonization by CRE / other multidrug-resistant organisms |  | Evidence of previous infection/ colonization by CRE / other multidrug-resistant organisms | At the moment of enrolment, was it known whether this patient had been previously colonized or infected with CRE, or other multi-drug resistant organisms, which include methicillin-resistant *Staphylococcus aureus*, vancomycin-resistant *Enterococci*, extended-spectrum beta-lactamase-producers), for which no time limit was given. There was no routine screening in most wards. |
| Antimicrobial exposure within 3 months before enrolment | colistin, aminoglycosides, quinolones, macrolides, cephalosporins, carbapenems, β-lactam+β-lactamase inhibitor, other beta-lactam antibiotics, antibiotics active against Gram-positive only, etc. |  |  |  |
| Clinical and microbiological characteristics of patients with *Enterobacterales* BSI | BSI source (urinary tract, pneumonia, intra-abdominal, intravascular catheter, other, unknown source), type of BSI acquisition (nosocomial, community-onset healthcare-associated, strict community-acquired), causative pathogen, presence and type of carbapenem resistance genes |  | Nosocomial BSI | Positive blood culture obtained from patients who had been hospitalized for 48 hours or longer^3,4^  If a patient was transferred from another hospital, the duration of inpatient stay was calculated from the date of the first hospital admission.^4^ |
|  |  |  | Community-onset healthcare-associated BSI | Positive blood culture obtained from a patient at the time of hospital admission or within 48 hours of admission if the patient fulfilled any of the following criteria^4^: 1) received intravenous therapy at home; received wound care or specialized nursing care through a health care agency, family, or friends; or had self-administered intravenous medical therapy in the 30 days before the bloodstream infection. Patients whose only home therapy was oxygen use were excluded; 2) attended a hospital or hemodialysis clinic or received intravenous chemotherapy in the 30 days before the bloodstream infection; 3) was hospitalized in an acute care hospital for 2 or more days in the 90 days before the bloodstream infection; 4) resided in a nursing home or long-term care facility. |
|  |  |  | Strict community-acquired BSI | Positive blood culture obtained at the time of hospital admission or within the 48 hours after hospital admission for patients who did not fit the criteria for a health care-associated infection.^4^ |

CRE, carbapenem-resistant *Enterobacterales*; MIC, minimum inhibitory concentration; CSE, carbapenem-susceptible *Enterobacterales*; BSI, bloodstream infection; SIRS, systemic inflammatory response syndrome.

**Table S2. Univariable analyses of risk factors for carbapenem-resistant *Enterobacterales* bloodstream infection among adult, hospitalized patients compared to matched controls with carbapenem-susceptible bloodstream infection, or without *Enterobacterales* infection**

| **All clinically relevant variables present before enrolment** | **Patients with CRE BSI (n=73) versus matched patients with CSE BSI (n=73)** | | **Patients with CRE BSI (n=73) versus matched patients without *Enterobacterales* infection (n=219)** | |
| --- | --- | --- | --- | --- |
|  | **IRR (95% CI)** | **P-value** | **IRR (95% CI)** | **P-value** |
| **Demographic information** |  |  |  |  |
| Age, years | 0.99 (0.97-1.02) | 0.639 | 1.03 (1.01-1.05) | 0.009 |
| Male sex | 1.13 (0.57-2.21) | 0.732 | 0.81 (0.47-1.39) | 0.447 |
| BMI, kg/m^2^ | 1.05 (0.98-1.14) | 0.182 | 1.00 (0.95-1.05) | 0.977 |
| BMI < 25 kg/m^2^ reference category |  |  |  |  |
| BMI 25-29 kg/m^2^ | 0.89 (0.41-1.93) | 0.764 | 1.22 (0.65-2.27) | 0.535 |
| BMI ≥ 30 kg/m^2^ | 1.05 (0.40-2.78) | 0.921 | 1.00 (0.46-2.16) | 0.990 |
| **Admission information** |  |  |  |  |
| Patient referral |  |  |  |  |
| Home (reference category) |  |  |  |  |
| Long term care facility | 3.31 (0.83-13.21) | 0.090 | 8.43 (2.10-33.88) | 0.003 |
| Another acute care hospital | 2.78 (0.81-9.55) | 0.105 | 5.30 (2.04-13.79) | 0.001 |
| Emergency admission | 0.67 (0.11-3.99) | 0.657 | 0.33 (0.06-1.83) | 0.206 |
| Length of hospitalization before enrolment ≥1 days^d^ | 2.00 (0.50-8.00) | 0.327 | 12.40 (1.40-109.53) | 0.023 |
| **Clinical characteristics** |  |  |  |  |
| Comorbidities |  |  |  |  |
| Charlson comorbidity index | 0.97 (0.82-1.14) | 0.683 | 1.09 (0.95-1.25) | 0.203 |
| Myocardial infarction | 1.00 (0.38-2.66) | 1.000 | 1.71 (0.71-4.11) | 0.230 |
| Congestive heart failure: NYHA grade ≥2 | 1.29 (0.48-3.45) | 0.618 | 0.95 (0.39-2.34) | 0.909 |
| Peripheral artery disease | 1.29 (0.48-3.45) | 0.618 | 0.87 (0.42-1.81) | 0.713 |
| Cerebrovascular disease | 1.00 (0.25-4.00) | 1.000 | 1.71 (0.49-5.97) | 0.402 |
| Dementia | 0.83 (0.25-2.73) | 0.763 | 2.57 (0.86-7.65) | 0.090 |
| Chronic pulmonary disease | 0.50 (0.17-1.46) | 0.206 | 0.54 (0.21-1.40) | 0.206 |
| Connective tissue disease | 2.00 (0.37-10.92) | 0.423 | 1.21 (0.37-3.99) | 0.752 |
| Ulcerative disease | 0.50 (0.05-5.51) | 0.571 | 0.64 (0.13-3.17) | 0.580 |
| Mild liver disease | 4.00 (0.45-35.79) | 0.215 | 1.78 (0.55-5.74) | 0.336 |
| Severe liver diseases | 1.00 (0.06-15.99) | 1.000 | 0.33 (0.04-2.88) | 0.318 |
| Diabetes mellitus without organ damage | 0.91 (0.39-2.14) | 0.827 | 1.39 (0.71-2.70) | 0.334 |
| Diabetes with target organ damage | 0.50 (0.17-1.46) | 0.206 | 1.00 (0.34-2.91) | 1.000 |
| Hemiplegia | 1.33 (0.30-5.96) | 0.706 | 3.00 (0.75-12.00) | 0.120 |
| Moderate or severe kidney disease | 2.75 (0.88-8.64) | 0.083 | 2.01 (0.86-4.74) | 0.108 |
| Metastatic solid tumor | 0.60 (0.14-2.51) | 0.484 | 1.16 (0.27-5.02) | 0.847 |
| Any tumor, not metastasic | 1.10 (0.47-2.59) | 0.827 | 1.65 (0.75-3.61) | 0.214 |
| Leukemia | 0.75 (0.17-3.35) | 0.706 | 1.17 (0.25-5.59) | 0.842 |
| Lymphoma | 1.00 (0.20-4.95) | 1.000 | 1.00 (0.28-3.57) | 1.000 |
| AIDS | - | - | - | - |
| HIV infection with <200 CD4/mm^3^ | - | - | - | - |
| Immunosuppression^a^ | 0.75 (0.32-1.78) | 0.514 | 1.26 (0.59-2.72) | 0.549 |
| Invasive procedures within 3 months before enrolment | 1.50 (0.67-3.34) | 0.321 | 5.87 (2.71-12.71) | <0.001 |
| Surgery during the previous month | 0.87 (0.32-2.41) | 0.796 | 1.74 (0.78-3.90) | 0.176 |
| Endoscopic procedure in the week before enrolment | 0.57 (0.17-1.95) | 0.372 | 1.36 (0.40-4.57) | 0.623 |
| **CRE exposure risk** |  |  |  |  |
| CRE exposure risk in community in the last six months |  |  |  |  |
| Travel abroad | 2.00 (0.18-22.06) | 0.571 | 0.35 (0.08-1.59) | 0.175 |
| Contact with pets at home | 1.00 (0.29-3.45) | 1.000 | 0.65 (0.28-1.51) | 0.315 |
| Frequent contact with livestock | - | - | - | - |
| Ambulatory contact with persons known to be colonized by CRE | - | - | - | - |
| CRE exposure risk in healthcare facility |  |  |  |  |
| Patient worked as healthcare worker or caregiver during last year | - | - | 1.50 (0.14-16.54) | 0.741 |
| Another patient/s with CRE in the same ward during present admission | 1.33 (0.56-3.16) | 0.514 | 1.27 (0.54-3.00) | 0.579 |
| Previous hospitalization during the last six months | 1.62 (0.81-3.23) | 0.174 | 2.78 (1.54-5.00) | 0.001 |
| Nursing home or other long term-care facility residency during the last six months | 1.14 (0.41-3.15) | 0.796 | 5.60 (1.70-18.41) | 0.005 |
| Chronic dialysis | 1.75 (0.51-5.98) | 0.372 | 2.27 (0.76-6.75) | 0.140 |
| Hemodialysis | 1.50 (0.42-5.32) | 0.530 | 3.50 (0.95-12.97) | 0.061 |
| Peritoneal dialysis | - | - | 0.72 (0.07-7.35) | 0.782 |
| Evidence of previous colonization/infection with CRE | 8.50 (1.96-36.79) | 0.004 | 54.00 (7.21-404.50) | <0.001 |
| Evidence of previous colonization/infection with other MDROs (MRSA, VRE, ESBL-producer) | 2.67 (0.71-10.05) | 0.147 | 4.72 (1.59-14.03) | 0.005 |
| **Antimicrobials exposure within 3 months before enrolment** | 1.45 (0.68-3.13) | 0.339 | 2.35 (1.25-4.40) | 0.008 |
| Colistin | 1.00 (0.25-4.00) | 1.000 | 5.00 (0.93-26.94) | 0.061 |
| Aminoglycosides | 1.40 (0.44-4.41) | 0.566 | 2.20 (0.79-6.09) | 0.131 |
| Quinolones | 1.50 (0.72-3.11) | 0.277 | 1.38 (0.75-2.55) | 0.298 |
| Macrolides | 0.67 (0.11-3.99) | 0.657 | 1.50 (0.27-8.19) | 0.640 |
| Cephalosporins, 1^st^/2^nd^ generation | 0.56 (0.19-1.66) | 0.292 | 1.69 (0.58-4.94) | 0.339 |
| Cephalosporins, 3^rd^/4^th^ generation | 1.50 (0.67-3.34) | 0.321 | 2.06 (1.02-4.14) | 0.043 |
| Carbapenems | 3.60 (1.34-9.7) | 0.011 | 4.36 (1.98-9.59) | <0.001 |
| β-lactam+β-lactamase inhibitor | 1.70 (0.78-3.71) | 0.183 | 3.13 (1.60-6.11) | 0.001 |
| Other beta-lactam antibiotics | 0.57 (0.17-1.95) | 0.372 | 6.42 (1.64-25.09) | 0.007 |
| Active against Gram-positive only | 2.67 (1.04-6.81) | 0.004 | 3.66 (1.76-7.64) | 0.001 |
| Other | 0.79 (0.36-1.73) | 0.549 | 1.37 (0.60-3.09) | 0.454 |

CRE, Carbapenem-resistant *Enterobacterales*; CSE, Carbapenem-susceptible *Enterobacterales*; BSI, bloodstream infection; IRR, Incidence rate ratio; CI, Confidence interval; BMI, body mass index; NYHA, New York Heart Association; AIDS, acquired immune deficiency syndrome; HIV, human immunodeficiency virus; MDROs, multidrug-resistant organisms; MRSA, methicillin-resistant *Staphylococcus aureus*; VRE, vancomycin-resistant *enterococci*; ESBL, extended-spectrum beta-lactamase.

-: The statistics could not be accurately estimated because no patients were positive in one of the groups or there were too few disconcordant pairs.

^a^ Immunosuppression was defined as the receipt of solid organ transplantation, bone marrow/stem cell transplantation, or immunosupressive drugs (including cancer chemotherapy, classic immunosuppresants, biologicals, steroids) last 3 months before enrolment, or with neutropenia (<500 cells/mm^3^) on enrolment.

**Table S3. Sensitivity analysis for missing data for “evidence available for previous colonization/infection with carbapenem-resistant *Enterobacterales*” comparing risk factors for carbapenem-resistant versus carbapenem-susceptible *Enterobacterales* bloodstream infections**

| **Variables** | **Worst-case scenario (n=146)** | | **Best-case scenario (n=146)** | | **Original model (n=140)** | |
| --- | --- | --- | --- | --- | --- | --- |
|  | **Incidence rate ratio (95% Confidence interval)** | **P-value** | **Incidence rate ratio (95% Confidence interval)** | **P-value** | **Incidence rate ratio (95% Confidence interval)** | **P-value** |
| Evidence of previous colonization/infection with CRE | 8.70 (1.96-38.65) | **0.004** | 7.53 (1.64-34.67) | 0.010 | 7.32 (1.65-32.38) | **0.009** |
| Carbapenems exposure within 3 months before enrolment | 3.06 (1.01-9.29) | **0.048** | 3.21 (1.07-9.62) | 0.037 | 2.76 (0.95-7.99) | 0.062 |
| Moderate or severe kidney disease | 2.83 (0.63-12.66) | 0.173 | 3.30 (0.79-13.75) | 0.101 | Not selected |  |

CRE, Carbapenem-resistant *Enterobacterales*; CSE, Carbapenem-susceptible *Enterobacterales*.

Bold texts indicate p value <0.05.

**Table S4. Sensitivity analysis for missing data for “evidence of previous colonization/infection with other multidrug-resistant organisms (methicillin-resistant Staphylococcus aureus, vancomycin-resistant enterococci, extended-spectrum beta-lactamase-producer)” comparing risk factors for carbapenem-resistant *Enterobacterales* bloodstream infection versus uninfected patients**

| **Variables** | **Worst-case scenario (n=292)** | | **Best-case scenario (n=292)** | | **Original model (n=288)** | |
| --- | --- | --- | --- | --- | --- | --- |
|  | **Incidence rate ratio (95% Confidence interval)** | **P-value** | **Incidence rate ratio (95% Confidence interval)** | **P-value** | **Incidence rate ratio (95% Confidence interval)** | P-value |
| Age, years | 1.04 (1.01-1.06) | **0.014** | 1.03 (1.01-1.06) | **0.015** | 1.03 (1.01-1.06) | **0.019** |
| Patient referral |  |  |  |  |  |  |
| Admission from home | Reference |  | Reference |  | Reference |  |
| Transfer from long term care facility | 7.12 (1.50-33.92) | **0.014** | 7.30 (1.52-34.95) | **0.013** | 7.19 (1.51-34.24) | **0.013** |
| Transfer from another acute care hospital | 5.27 (1.63-17.04) | **0.006** | 5.41 (1.70-17.28) | **0.004** | 5.26 (1.61-17.11) | **0.006** |
| Invasive procedures^a,b^ | 5.75 (2.16-15.36) | **<0.001** | 5.69 (2.12-15.27) | **0.001** | 5.66 (2.11-15.16) | **0.001** |
| Chronic hemodialysis | 8.38 (1.80-39.01) | **0.007** | 8.55 (1.84-39.77) | **0.006** | 8.59 (1.82-40.53) | **0.007** |
| Evidence of previous colonization/infection with other MDROs (MRSA, VRE, ESBL-producer) | 9.29 (2.55-33.82) | **0.001** | 10.02 (2.38-42.24) | **0.002** | 9.71 (2.33-40.56) | **0.002** |
| β-lactam+β-lactamase inhibitors exposure^b^ | 4.26 (1.84-9.87) | **0.001** | 4.00 (1.73-9.28) | **0.001** | 3.92 (1.68-9.13) | **0.002** |
| 3^rd^/4^th^ generation cephalosporins exposure^b^ | 2.49 (1.01-6.17) | **0.049** | 2.65 (1.08-6.47) | **0.033** | 2.75 (1.06-7.11) | **0.037** |

CRE, carbapenem-resistant *Enterobacterales*; BSI, bloodstream infection; MDROs, multidrug-resistant organisms; MRSA, methicillin-resistant *Staphylococcus aureus*; VRE, vancomycin-resistant *enterococci*; ESBL, extended-spectrum beta-lactamase.

Bold texts indicate p value <0.05.

^a^ central venous catheter, urinary catheter or mechanical ventilation

^b^ within 3 months before enrolment

**Table S5. Sensitivity analysis for nosocomial or community-onset healthcare-associated CRE BSI comparing risk factors for carbapenem-resistant versus carbapenem-susceptible *Enterobacterales* bloodstream infections**

| **Variables** | **The final model focusing on hospital-associated CRE BSI (n=129)** | | **The original model (n=140)** | |
| --- | --- | --- | --- | --- |
|  | **Incidence rate ratio (95% Confidence interval)** | **P-value** | **Incidence rate ratio (95% Confidence interval)** | **P-value** |
| Evidence available for previous colonization/infection with CRE | 7.14 (1.36-37.49) | **0.020** | 7.32 (1.65-32.38) | **0.009** |
| Carbapenems exposure within 3 months before enrolment | 3.23 (1.05-9.95) | **0.042** | 2.76 (0.95-7.99) | 0.062 |
| Chronic pulmonary disease | 0.21 (0.05-0.97) | **0.046** | Not included |  |

CRE, Carbapenem-resistant *Enterobacterales*; CSE, Carbapenem-susceptible *Enterobacterales*.

Bold texts indicate p value <0.05.

**Table S6. Sensitivity analysis for nosocomial or community-onset healthcare-associated CRE BSI comparing risk factors for carbapenem-resistant *Enterobacterales* bloodstream infection versus uninfected patients**

| **Variables** | **The final model focusing on hospital-associated CRE BSI (n=265)** | | **The original model (n=288)** | |
| --- | --- | --- | --- | --- |
|  | **Incidence rate ratio (95% Confidence interval)** | **P-value** | **Incidence rate ratio (95% Confidence interval)** | **P-value** |
| Age, years | 1.03 (1.00-1.06) | **0.050** | 1.03 (1.01-1.06) | **0.019** |
| Patient referral |  |  |  |  |
| Admission from home | Reference |  | Reference |  |
| Transfer from long term care facility | 8.46 (1.74-41.12) | **0.008** | 7.19 (1.51-34.24) | **0.013** |
| Transfer from another acute care hospital | 6.34 (1.76-22.88) | **0.005** | 5.26 (1.61-17.11) | **0.006** |
| Invasive procedures^a,b^ | 5.87 (2.08-16.60) | **0.001** | 5.66 (2.11-15.16) | **0.001** |
| Chronic hemodialysis | 7.75 (1.65-36.50) | **0.010** | 8.59 (1.82-40.53) | **0.007** |
| Evidence of previous colonization/infection with other MDROs (MRSA, VRE, ESBL-producer) | 3.78 (0.77-18.56) | 0.102 | 9.71 (2.33-40.56) | **0.002** |
| β-lactam+β-lactamase inhibitors exposure^b^ | 3.36 (1.45-7.82) | **0.005** | 3.92 (1.68-9.13) | **0.002** |
| 3^rd^/4^th^ generation cephalosporins exposure^b^ | 2.38 (0.88-6.44) | 0.087 | 2.75 (1.06-7.11) | **0.037** |
| Other beta-lactam antibiotics exposure^b^ | 7.03 (0.92-53.73) | 0.060 | Not included |  |

CRE, Carbapenem-resistant *Enterobacterales*; BSI, bloodstream infection; MDROs, multidrug-resistant organisms; MRSA, methicillin-resistant *Staphylococcus aureus*; VRE, vancomycin-resistant *enterococci*; ESBL, extended-spectrum beta-lactamase.

Bold texts indicate p value <0.05.

^a^ central venous catheter, urinary catheter or mechanical ventilation

^b^ within 3 months before enrolment

**References**

1. American College of Chest Physicians/Society of Critical Care Medicine Consensus Conference: definitions for sepsis and organ failure and guidelines for the use of innovative therapies in sepsis. *Crit Care Med* 1992; **20**: 864-74.

2. Charlson ME, Pompei P, Ales KL *et al*. A new method of classifying prognostic comorbidity in longitudinal studies: development and validation. *J Chronic Dis* 1987; **40**: 373-83.

3. Garner JS, Jarvis WR, Emori TG *et al*. CDC definitions for nosocomial infections, 1988. *Am J Infect Control* 1988; **16**: 128-40.

4. Friedman ND, Kaye KS, Stout JE *et al*. Health care--associated bloodstream infections in adults: a reason to change the accepted definition of community-acquired infections. *Ann Intern Med* 2002; **137**: 791-7.
